# Clinical Cases

**Published:** 1893-02

**Authors:** Erasmus E. Case

**Affiliations:** Hartford, Conn.


### CLINICAL CASES.

Erastus E. Case, M. D., Hartford, Conn.

### I. Picric Acid.

Mrs. W., aged twenty-seven years, a slight blonde lady of mild, yielding disposition, was delivered of her third child, without accident, on June 3d, 1886. Ten days later the albumen in the urine remained as copious as it was before her confinement. She had also suffered since the second day from a headache, which may be described as follows. She awakes every morning with a dull pain in one or both eyes. There is also a creeping or crawling sensation inside the skull. The pain increases in severity gradually, and extends through the whole head and down the nape of the neck, lasting all day. When the pain becomes severe it is pulsative in character, and worse by the slightest exertion of either the mind or body, even by turning the eyes. The pain passes off in the evening and she sleeps well through the night. After several useless prescriptions had been made ℞ *Pic-ac.*$^{12}$, *four doses, two hours' interval.*

This aborted the headache, which was then coming on, and it never returned. The albumen disappeared from the urine without further treatment.

### II. Lachnanthes Tinctoria.

January 18th, 1892.—Miss T., aged twenty-seven years, a brunette.

Muscular soreness, as if bruised.

Weariness, as if very tired.

Hands and feet cold.

Eyeballs sore to the touch; it hurts to turn them.

Photophobia.

The bridge of the nose feels as if it was pinched. ℞ *Lachnant.*$^{200}$, *four doses, two hours' interval.*

The peculiar symptom of the nose disappeared at once. A speedy restoration to health followed without further treatment.

This case attests the danger of ruining our materia medica by throwing out the chaff. The nasal symptom—as if the nostrils were pinched—is not found in Hering's *Guiding Symptoms.* Allen's *Encyclopedia* contains it with this explanatory foot-note: "The prover had fallen when a child and broken the nasal bones, which had partly suppurated and left an indentation.

### III. Thuja Occidentalis.

July 5th, 1890.—A Superintendent of Life Insurance Agencies, aged thirty-five years, has for a long time been troubled with vertigo while walking or riding in the cars, especially while walking after riding in the cars; worse by shutting the eyes, but better again on reopening them; better by lying down. With the vertigo there is a dull pain in the occiput. He is very nervous and apprehensive when about to transact any business, even when not important. Otherwise his health seems perfect. He denies having had gonorrhœa. ℞ *Thuja*$^{1m}$, *four powders, four hours' interval.*

No further treatment was needed.

### IV. Rhus Toxicodendron.

January 27th, 1892.—Robbie S., aged twelve years, dark brown hair and eyes, a hæmophilia, who would bleed to death from a slight cut unless pressure was applied. Six weeks previous to the date of the prescription he had been bruised on the left buttock. The blood had escaped into the cellular tissues, until the thigh was enormously distended. He became jaundiced while lying still waiting for the absorption of the blood.

Yesterday he had a chill. To-day temperature 102°.

Yellow skin.

Itching of the skin.

Stools in small white balls.

Urine contains clotted blood, also fresh blood as from a cut.

Doughy swelling about the left knee.

Stitches between the scapulæ upon every attempt to swallow, so that he eats and drinks nothing. ℞ *Rhus-t.*$^{1m}$, *four doses, two hours' interval.*

The stitching interscapular pains were relieved at once. Within two days the blood disappeared from the urine, and the stools became normal in color and character. In ten days he was restored to as good health as he ever enjoys.

### V. Lycoperdon Bovista.

February 6th, 1892.—Mrs. A., aged fifty-two years, a black-eyed and black-haired French Canadian, quite plethoric.

Frequent flushes of heat with vertigo. This condition is followed by chilliness, then sweat: worse when warm, either from the surrounding temperature or exertion, worse also at midnight.

Always is restless after midnight.

Palpitation of the heart, and sensation as if the heart were floating in water, when ascending or walking rapidly, sometimes also when too warm.

Much itching and burning of the skin, of feet especially: worse when warm and at night.

The hands are frequently numb. ℞ *Bov.*$^{45m}$ (*F.*), *one dose.*

This prescription relieved her so that she needed no further treatment.

### VI. Capsicum Annum.

June 18th, 1890.—Mrs. K., slender, dark hair and eyes, a nervous, fretful individual.

Facial neuralgia, beginning above the right temple and extending downward over the face, especially below the eye and on the right side of the nose. With the pain the face is sensitive to touch. Slight pressure will bring on the pain when it is absent. There is a circumscribed redness of the cheek with the pain. These sensations are described, viz.:—A crawling, ticking like a clock, burning like fire, like hot needles running through the face, like fine threads through the face drawn tightly. This neuralgia is an old enemy, and she has suffered for months at a time under allopathic treatment. ℞ *Capsicum*$^{1m}$, *four doses in one day.*

June 29th.—She has had no pain for more than a week. The soreness of the face is also gone. *No medicine.*

July 14th.—She has been overworking, and the pain returned two days ago, but less severely. ℞ *Capsicum*$^{40m}$ (*F.*), *one dose.*

She has had no neuralgia since then.

### VII.—Sabina.

August 9th, 1890.—Mrs. H., a slender brunette, married three years and childless, wishes some pediculated warts removed from the right side of the neck. I questioned her for other symptoms, but she resented the catechising, saying that she heard that I cured warts, and that was all she came to me for. I sat down and reasoned with her. After being told that I should prescribe for her and not for warts, and that if I succeeded in making her a well woman the warts would be gone, she gave these symptoms:

Frequently sick headaches—a pressive pain begins in the forehead and extends to the eyes and occiput, worse by lying down—cannot sleep with the headache, better by vomiting a sour fluid.

Bowels obstinately constipated; takes physic every night. Menses irregular in time, color, and quantity, frequently very profuse, always with severe pain from pubes to sacrum. Leucorrhœa thick, ropy, and albuminous. ℞. *Sabina*$^{1m}$, *four doses, one hour interval.*

September 2d.—Only one wart remains. The most troublesome one dropped off on the third day after the prescription. The menses came on without pain. *No medicine.*

May 18th, 1891.—Menstruation pretty comfortable until last month. The gums are spongy about decayed teeth and bleed freely. The warts are re-appearing. ℞. *Sabina*$^{45m}$ (*F.*), *one dose.*

This put an end to the dysmenorrhœa and warts. The headaches were already mitigated.

### VIII.—Graphites. Sulphur.

June 20th, 1890.—A servant girl aged thirty-five years has had eczema upon the left wrist for six weeks. It is covered by a thick brown crust, which cracks and peels off at intervals of from one to three days, leaving a raw surface, from which a sticky moisture exudes in drops. This soon forms another crust. It has intense burning and itching, worse at night and from bathing it. The feet frequently burn at night so that she uncovers them. She is constipated and the stools are large and hard. The menses come early and are painful. ℞ *Graph.*$^{1m}$, *four doses, one hour interval.*

July 6th.—A severe aggravation followed the prescription, and spots of eczema appeared upon the back. *No medicine.*

July 13th.—The eczema has gone from the back, but is showing on the abdomen. The wrist has less itching and moisture; the crust is thin and losing its tendency to crack. The bowels are regular and stools natural. The eyes are full of tears; the vision has become so weak that she cannot see to read. (A pathogenetic symptom.) *No medicine.*

July 20th.—The wrist is still improving. No eczema now on body. The eyes are even weaker with more lachrymation. *No medicine.*

August 3d.—The spot of eczema is larger, with a very thin scale and no moisture. It becomes red, painful, and sore if it is wet. It has voluptuous itching in the evening when heated. The eyes are about well. ℞ *Sulph.*$^{1m}$, *one dose.*

September 7th.—Immediate improvement followed. Two weeks ago ulceration occurred about the nail of the second finger of the left hand. No sign of the eczema since. *No medicine.*

### Discussion.

Dr. W. Wesselhœft—Please give the complexion and temperament of the woman who was cured with Capsicum.

Dr. Case—Dark complexion, hair and eyes; irritable and fretful in disposition.

Dr. W. Wesselhœft—I have always thought of Capsicum in flabby, relaxed women, and very rarely in thin, nervous women.

Dr. Case—If she gains in flesh another year as rapidly as during the last six months, she will be somewhat like the typical Capsicum patient.

